# A maxillary center incisor with three independent roots and three root canals: A case report

**DOI:** 10.1097/MD.0000000000021761

**Published:** 2020-08-14

**Authors:** Jia Wang, Wenyi Zhang, Lei Zhou

**Affiliations:** a Department of Endodontic; b Department of Periodontology and Oral Mucosal Diseases; c Department of Endodontic, Qingdao Stomatological Hospital, Qingdao, China.

**Keywords:** maxillary center incisor, root canal morphology, the cone-beam computed tomography (CBCT), three root canals

## Abstract

**Rationale:**

Three root canals (mesiobuccal, distobuccal and palatal) are rarely found (frequency <1%) in the maxillary central incisor even though root canal morphology in maxillary premolars is highly variable. Therefore, research papers showed that dentists can easily miss the root canals in diagnosis and inflammatory diffusion; which could cause unsuccessful root canal treatment leading to various possible infections and no change in original inflammations. In this report, the diagnose and clinical management of an unusual case of a maxillary center incisor with three independent roots and three root canals is presented, along with a demonstration of using CBCT (Cone Beam Computed Tomography) and collaborate with other departments to successfully accomplish an accurate diagnosis of the morphology and quantity of the root canal system.

**Patient concerns:**

The patient was referred to clinic for his repeatedly abscessed in the gums of the left upper central incisor.

**Diagnoses:**

Based on clinical and radiographic evidences, the patient was tentatively diagnosed with a chronic periapical periodontitis for #21 tooth.

**Interventions:**

The patient was performed with the conventional root canal treatment and then clinical observed.

**Outcomes:**

At the second visit after 7 days, the patient was not sensitive to percussion. After operation for 3 months, and found that the sinus opening had not healed. Then, the patient was undergone with the periodontal flap surgery to remove root infection for 2 weeks.

**Lessons:**

From this clinical case, the lesson learned is that the previous clinical experiences cannot be used to make judgments or decisions; it requires specific analysis from the information gathered through CBCT(Cone Beam Computed Tomography)and the cooperation between different departments to come up with a responsible decision. In any stomatological hospitals, due to the large number of departments and the strong specialized focuses for each department; it is very important to encourage and support the cooperation between the departments, to limit any judgment bias due to lack of knowledge and maximize each department's strengths.

## 1 Introduction

Root canals treatment is optimal choice for the endodontic and periapical diseases. However, many of formidable challenges were found in the treatment of root canals owing to morphology variations of root canals. It is of utmost importance of a thorough knowledge of root canal anatomy and morphology, and accomplishment of the exhaustive removal of bacteria from the root canal system and their by-products.^[1,2]^ A number of studies of root canal anatomy have demonstrated the variation of maxillary lateral incisor or premolars. The maxillary central incisors are frequently to be reported to have only one root and one canal, and some clinical reports described a maxillary central incisor with two canals or two root.^[3–5]^ The maxillary central incisor with three or four root canals were very rarely been reported.^[6,7]^ Here we represent a case of a maxillary center incisor with three independent roots and three root canals.

## 2 Case report

**Figure 1:**
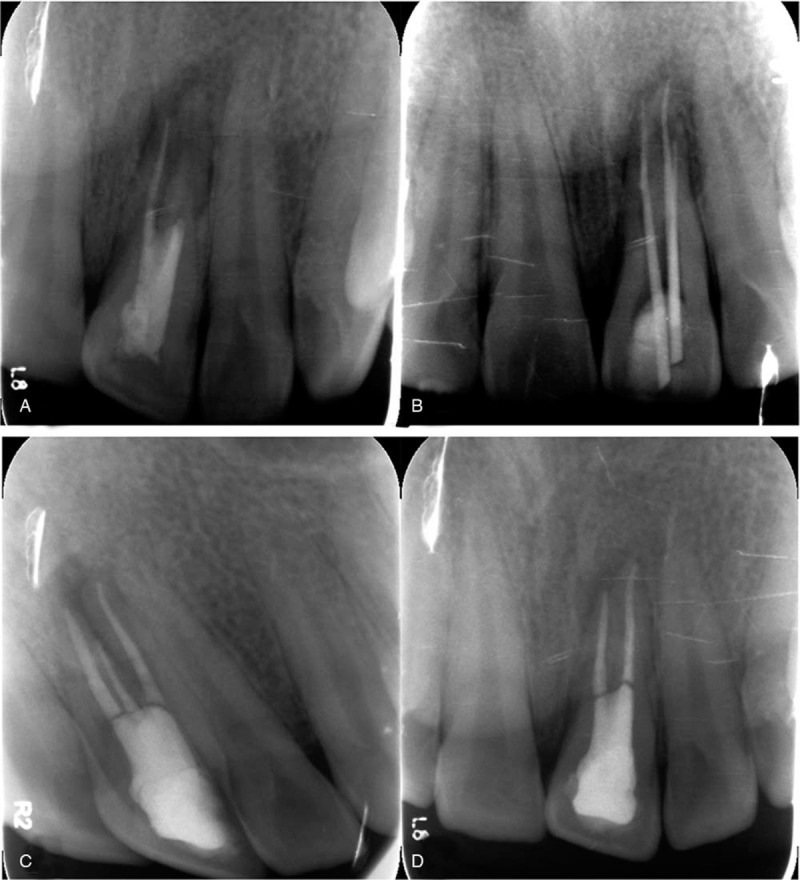
(A) The preoperative, (B) intraoperative, and (C and D) postoperative radiograph of #21 tooth.

A 20-year-old male patient was referred to clinic for his repeatedly abscessed in the gums of the left upper central incisor. From detailed dental history given by himself, it emerged that the patient suffered an injury by collision with basketball at 10 years ago, and was treated in other hospital but no obviously recovery obtained. Moreover, he had no previous history of systemic diseases and any known allergy. The present intraoral examination demonstrated that #21 tooth crown was normal, and a sinus tract opening was seen on upper #21 tooth, with purulent fluid when squeezing. Meanwhile, there was no obvious dental tissue disorder in #21, and there were periodontal pockets of 6-mm with lip side and of 10-mm with palatal side. No electrical activity was examined, and percussed pain was tender with a degree of “+.” The MinRay X-ray (Soredex, Nahkelantie, Finland) examination showed low density areas in #21 tooth apex and high density areas in root canals, whose medical images were not clear (Fig. 1). Based on clinical and radiographic evidences a tentative diagnosis of chronic periapical periodontitis for #21 tooth was made.

Thus, treatment planning was to first perform the conventional root canal treatment and then expose to clinical observation. If not successfully, the periodontal flap surgery would be performed. Lastly, the tooth would be extracted.

**Figure 2:**
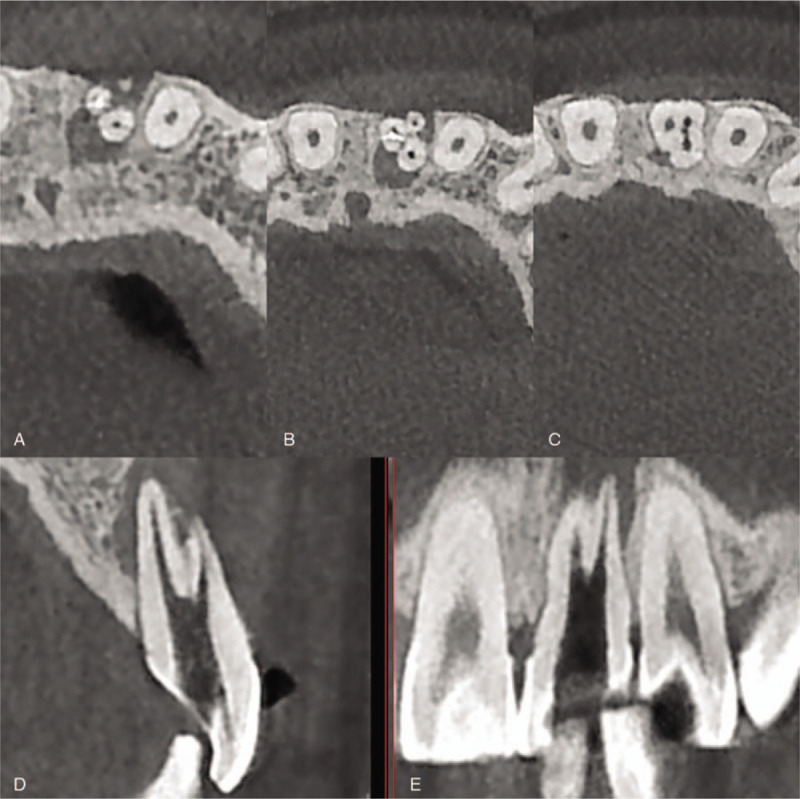
The cone-beam computed tomography (CBCT) scan of root canals, which distanced from root apex respectively (A) 3 mm, (B) 6 mm, and (C) 9 mm; (D) the sagittal view and (E) axial view of CBCT scan.

Informed consent was obtained from the patient for root canal treatment of the involved teeth. Patient has provided informed consent for publication of the case. Three canal orifices in #21 were uncovered in exposure to microscope (OMS2355, Zumax, Suzhou, China) after uncovering the roof of pulp chamber completely and removing the previous fillings. Moreover, the cone-beam computed tomography (CBCT; NewTom VG, QR SRL, Verona, Italy) images revealed that there were three independent roots with three root canals, which distanced from root apex were respectively 3, 6 ,and 9 mm (Fig. 2). Working length (WL) was established by electronic apex locator (Raypex6, VDW, Munich, Germany) and the Protaper Universal (Dentsply Maillefer, Ballaigues, Switzerland) and 17% viscous ethyleneaminetetraacetic acid (EDTA; Pulpdent Corp., Oakland) gel were respectively prepared up to #F2 by Nickel-titanium root canal preparation system (X-Smart, Dentsply). During preparation of root canal, 3% hydrogen peroxide (H_2_O_2_; LIRCON, Shandong, China) and 5.25% sodium hypochlorite (NaClO; LIRCON) were used as the irrigants and the root canals were rinsed for 3 three minutes. The access cavity was filled with Calcium hydroxide paste (Pulpdent Corp.) and temporarily restored with Caviton (GC Corp., Tokyo, Japan).

**Figure 3:**
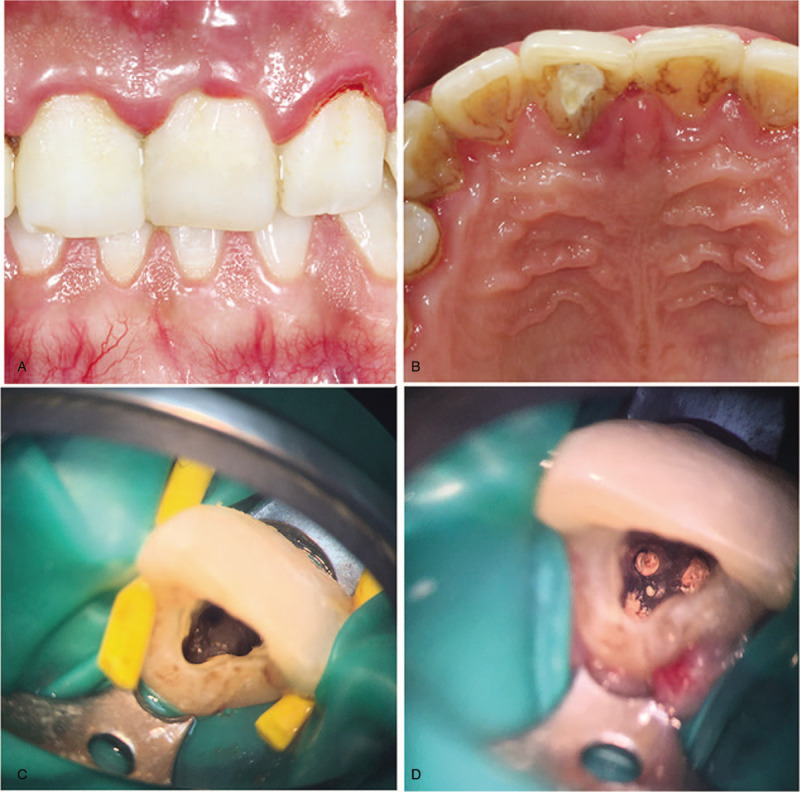
Intraoral photographs: (A) anterior view; (B) palatal side view; (C) intraoperative view; (D) postoperative view.

**Figure 4:**
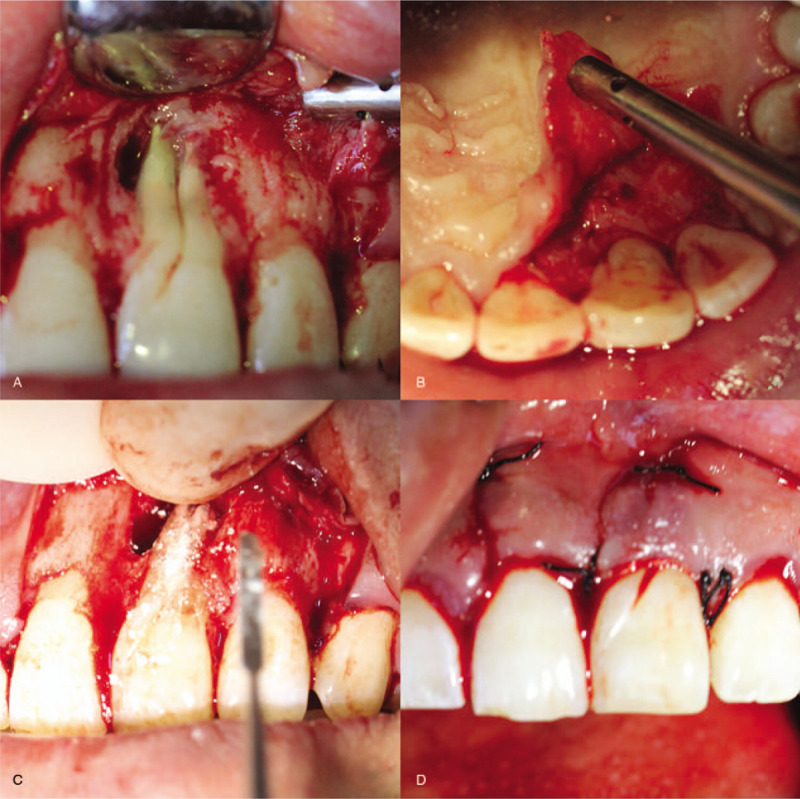
Intraoral photographs of follow-up visit after operation between 3 months. (A) Anterior view; (B) palatal side view; (C) anterior view after MTA filling; (D) anterior view after suture.

At the second visit after 7 days, the patient was not sensitive to percussion, and warm gutta-percha obturation technique was employed. Canals were filled with an apical size of #30/0.04 and a nanofilled composite resin (Filtek Z350 3 M ESPE, St Paul, MN), and sealed with AH Plus (Dentsply) (Fig. 3). The patient was visited for 2 times after operation for 3 months, and found that the sinus opening had not healed. After consultation with the periodontal mucosa doctors, the patient was undergone with the periodontal flap surgery to remove root infection and the uneven groove on the labial surface was filled with mineral trioxide aggregate (MTA; Dentsply). Palatal root surface was scraped and smoothed (Fig. 4).

## 3 Discussion

On the basis of depth of enamel invagination inside the tooth, the dens invaginatus were frequently classified into 3 types demarcated by Oehlers.^[8]^ For instance, type 1, saclike invagination was limited to the coronal part, and not extended beyond the cementoenamel junction (CEJ); type 2, invagination was advance beyond the CEJ but not joined the periodontal ligament; type 3a, invagination reached through the root surface and was linked together with periodontal ligament laterally; type 3b, invagination was arrived to the root and communicated with the periodontal ligament at the apical foramen.^[9]^ The maxillary central incisor in this case was regarded as Oehlers type 3a. The root is divided into three parts by the longitudinal fissure of the buccal and the lingual sides, which extends from the neck of the tooth to the root furcation, forming three independent roots, and each root has a single root canal. Although it is extremely rare to find the more than an additional root canal in the maxillary central incisor,^[9,10]^ an astute clinician needs to be conscious of unexpected root canal morphology when performing root canal therapy. It is difficult to be completely cured only with endodontic therapy if root infection extends to the periapical area.

In addition to a proficient knowledge of pulp anatomy and skilled operation, some of the commercially available instruments and materials are very helpful for the clinicians to accomplish their goals. CBCT is a relatively new means to display an individual tooth or dentition with reference to surrounding skeletal tissues in three dimensions, showing the anatomy of the teeth and providing more comprehensive imaging information for root canal treatment.^[1,11]^ In this case, CBCT was advocated to confirm the quantity of root canals in the anterior tooth, the root groove and the absorption range of the apical periodontal bone.

As the close links between root groove and the absorption of the apical periodontal bone, the groove was performed with flapping and filled with MTA after accomplished the canal treatment. MTA has been used in dentistry as a Portland cemented-based material for over 20 years. MTA has been reported to possess good biocompatibility and bioactivity, for which it popular used in pulp capping, pulpotomy, apexification, and as root end filling material.^[12]^ Moreover, it can promote the formation of alveolar bone and cementum and the reconstruction of periodontal ligament for its osteogenic induction. Furthermore, recent studies proved that MTA exhibited antibacterial activity, which have a crucial influence on the success of root canal treatment.^[13,14]^

The factors that lead to root anomalies might be complicated, which may be related to the separation of epithelial septum and ectodermal mesenchymal cells due to accidental factors during the development of tooth germ epithelial root sheath. Such factors have been reported to be involved in the development of tooth roots, as transforming growth factor β (TGF-β), bone morphogenetic protein (BMP), fibroblast growth factors (FGFs), Sonic Hedgehog (Shh), Notch, Msxl/2, nuclear factor I-C (Nfic), and so on.^[15,16]^ And any anomaly of these factors may lead to the root deformity.

The present report represents the diagnose and clinical treatment of an unusual case of a maxillary center incisor with three independent roots and three root canals, which emphasizes the significance of precise diagnose and careful clinical for endodontic practice.

## Author contributions

**Writing – original draft:** Jia Wang.

**Writing – review & editing:** Wenyi Zhang, Lei Zhou.
